# Construction of Effective Minimal Active Microbial Consortia for Lignocellulose Degradation

**DOI:** 10.1007/s00248-017-1141-5

**Published:** 2018-02-01

**Authors:** Pilar Eliana Puentes-Téllez, Joana Falcao Salles

**Affiliations:** 1 Microbial Community Ecology, GELIFES — Groningen Institute for Evolutionary Life Sciences, University of Groningen, Nijenborgh 7, 9747 AG Groningen, The Netherlands; 2 Present Address: Department of Biology, Institute of Environmental Biology, Ecology and Biodiversity Group, Padualaan 8, 3584 CH Utrecht, The Netherlands

**Keywords:** Lignocellulose, Degradation, Microbial consortium, Functional diversity

## Abstract

Enriched microbial communities, obtained from environmental samples through selective processes, can effectively contribute to lignocellulose degradation. Unfortunately, fully controlled industrial degradation processes are difficult to reach given the intrinsically dynamic nature and complexity of the microbial communities, composed of a large number of culturable and unculturable species. The use of less complex but equally effective microbial consortia could improve their applications by allowing for more controlled industrial processes. Here, we combined ecological theory and enrichment principles to develop an effective lignocellulose-degrading minimal active microbial Consortia (MAMC). Following an enrichment of soil bacteria capable of degrading lignocellulose material from sugarcane origin, we applied a reductive-screening approach based on molecular phenotyping, identification, and metabolic characterization to obtain a selection of 18 lignocellulose-degrading strains representing four metabolic functional groups. We then generated 65 compositional replicates of MAMC containing five species each, which vary in the number of functional groups, metabolic potential, and degradation capacity. The characterization of the MAMC according to their degradation capacities and functional diversity measurements revealed that functional diversity positively correlated with the degradation of the most complex lignocellulosic fraction (lignin), indicating the importance of metabolic complementarity, whereas cellulose and hemicellulose degradation were either negatively or not affected by functional diversity. The screening method described here successfully led to the selection of effective MAMC, whose degradation potential reached up 96.5% of the degradation rates when all 18 species were present. A total of seven assembled synthetic communities were identified as the most effective MAMC. A consortium containing *Stenotrophomonas maltophilia*, *Paenibacillus* sp., *Microbacterium* sp.*, Chryseobacterium taiwanense*, and *Brevundimonas* sp. was found to be the most effective degrading synthetic community.

**Electronic supplementary material:**

The online version of this article (10.1007/s00248-017-1141-5) contains supplementary material, which is available to authorized users.

## Introduction

The biological degradation of lignocellulosic waste materials for subsequent energy production is considered a very promising and sustainable way to supply energy demands. For instance, lignocellulose agrowaste, such as straw and bagasse from sugarcane production, can potentially be used in industry, generating a wide range of value-added bioproducts (e.g., biogas, enzymes, antioxidants) and biofuels [1]. This degradation process relies on the breakdown of lignocellulose—composed of hemicelluloses, cellulose, and the recalcitrant aromatic compound, lignin—through chemical, enzymatic, or thermomechanical processes that convert the polysaccharides into their constituent sugars [2].

The use of microbial consortia is considered an effective and sustainable way of promoting lignocellulose degradation, demonstrating enhanced degradation potential when compared to monoculture approaches, i.e., individual isolates [3–5]. Microbial consortia can be defined as communities ranging from two species communities to undefined, multi-species aggregations, where microorganisms use diverse mechanisms involving multiple complementary enzymes, particularly glycoside hydrolases (GHs), to deconstruct hemicellulose and cellulose. Lignin depolymerization on the other hand is achieved by using peroxidases and laccases [6]. Thus, the degradation of lignocellulose by microbial communities involves several complex and sometimes overlapping mechanisms, which are in general the result of a common effort by the members of the microbial community, due to resource complementarity.

Relationships among species diversity, stability, and function have been central topics in microbial ecology for several decades. Mounting evidence has demonstrated that the direct and positive relationship between function and diversity [7, 8] is often driven by the high complementarity between species, where higher productivity is observed in functionally diverse communities, as determined by their metabolic potential [9, 10]. In addition, ecological evidence supports the contention that microorganisms in a community depend on the activity of other microorganisms to grow, adapt, and reproduce [11–13]. To do so, they make use of complementary mechanisms involving the acquisition and exchange of metabolites to efficiently extract available energy resources. Thus, mixed populations are expected to perform functions that are difficult or even impossible for single species [14] while dealing with potential environmental fluctuations. These ecological principles underlie the higher effectiveness of lignocellulose-degrading microbial consortia and are currently being used to engineer microbial consortia able to effectively perform a range of processes [15].

The enrichment culture technique is a powerful tool to obtain microbial consortia with desired degradation properties [16, 17] because microbial consortia obtained with this method are closer to those functioning in nature [16]. Thus, in the last decades, several lignocellulose-degrading microbial consortia enriched from environmental samples have been identified and functionally characterized [18–22], revealing that functional redundancy is present in these systems [23]. Up to now, it is clear that microbial consortia production systems must account for the environmental relationships, distribution, abundance, and functional diversity of the participating members and their specific role during degradation [24].

Even though the analysis of enriched communities from environmental samples reveals the dominance of few bacterial phyla (e.g., *Proteobacteria*, *Firmicutes*, *Chloroflexi*, and *Bacteroidetes*), the number of ecological dominant species (culturable and unculturable) remains high, i.e., > 20 identified strains [18, 25–29]. The complexity of such systems can make it difficult to disentangle the interactive network that ultimately drives the degradation process. Importantly, the lack of knowledge on the interactions between these large numbers of species during degradation hampers the upscaling of the consortia to an industrial system designed to obtain lignocellulose biodegradation and derived products like biofuels. In addition, the fact that not all effective strains from the enrichments can be isolated and grown in the lab limits their application for industrial purposes. Thus, designing an effective degrading consortium harboring a reduced number of culturable members could be advantageous, enabling a full characterization of the structural composition, the degradative power, and the interactive roles of degradation players. In other words, a better understanding of the degradation process could pave the way for a more efficient breakdown of lignocellulose for environmental and commercial purposes.

In order to obtain a minimal active microbial consortia (MAMC) from environmental samples, we used an ecologically driven, reductive-screening approach (reducing the number of species throughout the investigation), starting with the isolation of lignocellulose-degrading strains obtained by selection through an enrichment process of soil bacteria grown in sugarcane-biowaste lignocellulose substrates, followed by molecular phenotyping, identification, and metabolic characterization. Based on the metabolic characterization, 45 bacterial strains were classified as belonging to four functional metabolic groups. A set of 18 strain representatives of these groups were further used to construct a total of 65 synthetic communities containing five species each (65 compositional replicate MAMCs). Thus, MAMCs varied in their functional diversity, as determined by the number of functional metabolic groups, as well as in metabolic and degradation potential, while remaining constant in species richness. The MAMCs were evaluated for their degradation capacity, under the hypothesis that MAMC with higher functional diversity would lead to higher degradation rates.

## Materials and Methods

### Isolation and Maintenance of Strains

In order to obtain bacteria with high lignocellulose degradation capacity, we performed an enrichment experiment [28–30] using two sugarcane related substrates—bagasse (B) and straw (St)—and a soil inoculum obtained from a sugarcane plantation, which generated a total of 18 flasks including controls with substrate and no inoculum. Briefly, 10 g of soil inoculum was used to prepare a soil suspension by adding to 90 mL of sodium chloride 0.90% and 10 g of sterile gravel in 250-mL flasks. After shaking for 1 h at 250 rpm at room temperature (20 °C), aliquots of 250μL were inoculated to triplicate 100-mL flasks containing 25 mL of mineral salt sterile medium (MSM) [30] with 1% of the lignocellulose sterile substrate. Cell density was controlled microscopically at regular time intervals and when cultures reached 10^−9^ cells/mL, an aliquot of 25 μL of culture was transferred into 25 mL of fresh medium. After ten transfers and after the final flasks reached 10^9^ cells/mL, the enrichment process was stopped. A 2-mL sample from several flasks of lignocellulose-enriched soil community’s final transfer (T10) was then used for colony isolation (for the enrichment experiment, see supplementary Fig. S1(a)). A homogenized sub-sample of 1 mL was used for serial dilutions in NaCl 0.85% until 10^5^ and plated onto minimal nutrient medium agar plates (Reasoner’s 2A agar, R2A) (Becton Dickenson, Cockeysville, MD) in triplicates. Colonies were chosen according to morphological uniqueness via visual inspection and were further purified using several transfer steps and maintained in R2A agar. For long-term preservation and further studies, fresh biomass of the isolates obtained with LB broth (72 h of growth) was suspended in 20% glycerol and stored at − 20 °C.

### Genotypic Differentiation Using BOX-PCR (Molecular Fingerprinting)

We performed colony BOX-PCR in order to compare the genomic profiles of all isolated colonies, and select for individual strains. BOX-PCR was performed with the primer A1R (CTACGGCAAGGCGACGCTGA) and the following PCR amplification conditions: 95 °C for 2 min; 35 cycles of 94 °C for 30s, 50 °C for 1 min, and 65 °C for 8 min; and a final extension step at 65 °C for 16 min [31]. PCR products were then run in 1.5% agarose gel and further analyzed (cluster analysis of BOX-PCR pattern) in GelCompar II (Applied Maths) using Dice coefficient and the UPGMA clustering. Isolates with a > 95% homology were assigned as belonging to the same strain.

### Phylogenetic Identification of Isolates by Partial 16S rRNA Gene Sequencing

The DNA extraction from purified cultures of a total of 96 representative strains that belonged to unique BOX groups was performed with the UltraClean® Microbial DNA Isolation Kit (MoBio® Laboratories Inc., Carlsbad, CA, USA) according to the manufacturer’s instructions. We assigned an ID number to each strain (ID strain number 1 to 96) for identification purposes during the study. Bacterial 16S rRNA genes were amplified using primers B8F (5-AGAGTTTGATCMTGGCTCAG-3′ [32]) and U1406R (5′-ACGGGCGGTGTGTRC-3′ [33]). PCR reactions were done in a 50-μL reaction mixture following the protocol of Taketani et al. [34]. PCR products were sequenced by Sanger technology (LGC Genomics, Germany). All resulting chromatograms were analyzed and trimmed for quality using the Lucy algorithm (http://rdp.cme.msu.edu/). Taxonomic assignment of the sequences (> 99% identity) was done using BLAST-N against the National Center for Biotechnology Information (NCBI) database (http://blast.ncbi.nlm.nih.gov/Blast.cgi) and confirmed at RDP (Ribosomal database project).

### Enzymatic Activities Related to Hemicellulose and Cellulose Degradation

In order to determine distinct enzymatic degradation potentials of the strains identified by BOX-PCR and 16S rRNA gene sequencing, we quantified the enzymatic activities related to hemicellulose and cellulose breakdown during growth in MSM media with 1% of the specific lignocellulosic substrate from which they were originally obtained. Microbial cells plus substrate from 2-mL samples were harvested by centrifugation for 5 min at 10000 rpm after 72 h of growth. The supernatant (secretome) was recovered and tested for enzymatic activity in triplicates using MUF-ß-D-xylopyranoside, MUF-ß-D-mannopyranoside, MUF-ß-D-galactopyranoside, MUF-ß-D-cellobioside, and MUF-ß-D-glucopyranoside as substrates. For the reaction, 10 μL of MUF-substrate (10 mM in dimethyl sulfoxide), 15 μL of Mcllvaine buffer (pH 6.8), and 25 μL of each supernatant were mixed and incubated at 28 °C for 45 min in the dark. The reaction was stopped by adding 150 μL of 0.2 M glycine-NaOH buffer (pH 10.4). Fluorescence was measured at an excitation of 365 nm and emission of 445 nm. The negative controls consisted in sterile PCR water and a mixture without the MUF substrates. Enzyme activities were determined from the fluorescence units using a standard calibration curve built with glycine buffer (pH 10.4) and expressed as rates of MUF production (μM MUF per min at 28 °C pH 6.8).

### Phenotypic Diversity (Biolog GEN III Plates)

The metabolic profiles of 45 enzymatically active selected strains were monitored during growth on 71 energy sources of GEN III Biolog plates (Biolog Inc., Hayward, CA). The absorbance values at 590 nm were measured using a microplate reader (VersaMax microplate reader; Molecular Devices Corp.). For each strain, colonies obtained on R2A agar were pulled into inoculation fluid (IF-B) at an optical density (OD_590_) of 0.03. After 2 h of starvation in the IF-B fluid, 100 μL was transferred to each well of the GEN III plates including one blank. The plates were incubated at 28 °C and read every 12 h until 72 h of growth. The normalized sum of all measured time points for the well was used to calculate the area under the curve for each carbon source. These data were used to generate a principal component analysis (PCA) using Canoco v5.0 [35], which allowed the classification of strains into functional metabolic groups (FMG), by calculating the similarities in metabolic profiling across all 71 substrates.

### MAMC

A total of 18 strains representing all FMG were used to create compositional replicates of five-species MAMC. Specifically, a total of 65 different synthetic communities were created by combining members of two, three, or all four FMG (Table 1), thus generating MAMC with different levels of functional diversity (two, three, or four FMG) but similar levels of species richness. For each level of functional diversity, we prepared three to four compositional replicates of each synthetic community, i.e., using the same number of species from the same FMG but different species, leading a total of 65 synthetic communities. The use of compositional replicates allowed for proper quantification of functional diversity on the substrate’s degradation while controlling for the influence of species identity.

**Table 1:**
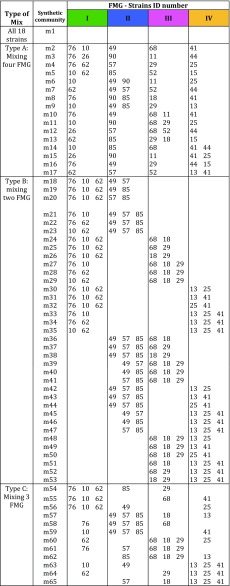
Description of the MAMC according to the number of functional metabolic groups. Type of mix according to the number of functional metabolic groups (FMG) combined (FMG I, FMG II, FMG III, FMG IV) and ID number of strains used in each synthetic community per functional group. All synthetic communities were at least three compositional replicates. m: identification of the synthetic community. For strain identification, see Table 2

After selecting the composition of each one of the 65 MAMCs, these were constructed by mixing all strains in equal concentrations. Briefly, we grew the strains in R2A broth during 40 h (180 rpm, 28 °C) and adjusted each strain to an OD_590_ of 0.02 in NaCl 0.85%. An aliquot of 125 μL of each diluted strain (approximately 1 × 10^5^ cells/mL, as determined by plate counting) was inoculated into 25 mL of MSM medium with 1% of straw (total cell concentration of 5 × 10^5^ cells/mL in 25 mL). In order to generate a control with maximum functional diversity and species richness, we also created a synthetic community containing all selected strains (18 in total). For this, we inoculated approximately 3 × 10^4^ cells/mL of each strain. All consortia were incubated at 28 °C 180 rpm for 96 h.

### FTIR Analysis

For the calibration set, pure cellulose (microcrystalline powder), hemicelluloses (xylan from birch wood), and lignin (hydrolytic) powders were obtained from Sigma-Aldrich Canada Ltd. (St. Louis MO) and were subsequently mixed in different proportions [36] to determine the relationship between their respective quantity in the synthetic community and representative Fourier transform infrared spectroscopy (FTIR) spectra [36]. Particle size of both the calibration set and samples was defined with a 106 μm sieve. Spectra were recorded using a Perkin-Elmer VATR Two spectrometer (Waltham MA USA) in the wavenumber range of 800–1800 cm^−1^ with a resolution of 4 cm^−1^ under ambient atmosphere at room temperature and in triplicates. The spectra were integrated and baseline corrected using the Spectrum ™ software. The analysis was performed using the Unscrambler X (Camo Software Oslo Norway). A 5-point Savitzky–Golay smoothing algorithm was applied to the calibration set’s spectra and used to predict concentrations in the samples (using partial least squares (PLS) regression). The predicted composition of each sample obtained with PLS was expressed as the percentage of degradation (from the initial amount; %D) and was calculated for each lignocellulose fraction of the lignocellulose as follows: %D = [(a − b) / a] × 100; where a = percentage of the fraction in the substrate before incubation; b = percentage of the fraction in the substrate after incubation.

### Functional Diversity Measurements

The community niche (CN) of a given MAMC was obtained based on the performance of each species on each one of the 71 carbon sources from the Biolog GEN III plate [9]. The CN value corresponds to the sum of the best performances per carbon source found in each synthetic community. Furthermore, we used the metabolic potential of each strain, also based on Biolog data, to calculate the functional attribute distance (FAD), used as a proxy of functional diversity [37, 38]. FAD was calculated by using Euclidean distance to calculate the pairwise distance between species (Function dist{} implemented in RStudio 1.0.136 [39].

## Results

### Isolation and Identification of Enriched Bacterial Strains

Samples from the end-point populations (Transfer 10) of all the cultures with substrate (including the controls) were diluted and plated onto R2A agar. A total of 157 isolates (up to 16 colonies per flask) were obtained from the last three plated dilutions. Molecular characterization of the purified strains using BOX PCR revealed a total of 96 unique groups across all samples, which were identified by sequencing of the 16S rRNA gene in 72 bacterial species (see Table S1 supplementary information for identity and GeneBank accession numbers).

### Enzymatic Degradation Potentials

The enzymatic degradation potential of 72 strains was measured after 72 h of growth under the enriched correspondent substrate. Results did not have a correlation with the substrate type in all MUF substrates (*p* > 0.05). The highest enzymatic activity in all secretomes was observed in the ß-d-mannopyranoside activity (related to hemicellulose degradation) with an averaged 1.17 μM MUF/min followed by 0.2 μM MUF/min of ß-d-cellobioside (involved in cellulose degradation) and 0.12 μM MUF/min of ß-d-xylopyranoside (as part of hemicellulolitic activities). Based on enzymatic activity potential, we selected a total of 25 strains from bagasse and 20 strains from straw (45 strains) (Fig. S2 Supplementary information; identity of these 45 strains can be found in Table S1).

### Metabolic Diversity and Construction of Consortia

**Fig. 1:**
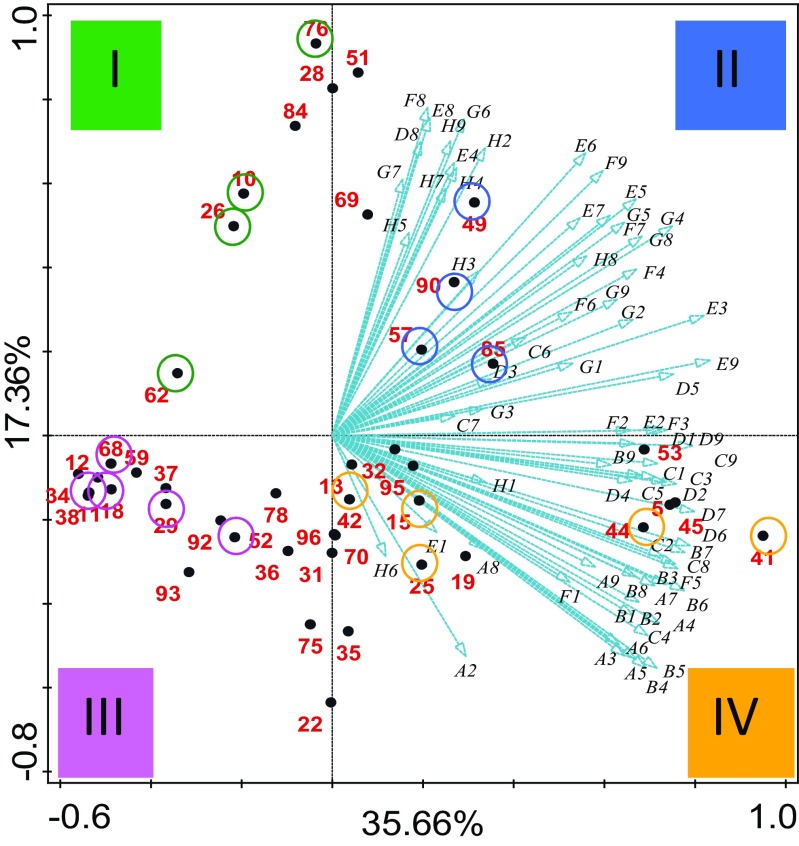
Principal component analysis (PCA) of the individual bacterial stains, which led to their classification into four functional metabolic groups (FMG), according to the four sections in the plot (I, II, III, IV), as indicated by the different colors. The graph is based on the metabolic potential of the individual strains, as determined by measuring the area under the curve after 72 h of incubation of the 45 selected strains in Biolog GEN III plates. Blue vectors represent the different energy sources of Biolog GEN III plates. Bigger-colored circles indicate the 18 selected strains that were used to construct the MAMC and their respective FMG

In order to construct the MAMC, we first characterized the metabolic profile of the 45 selected strains using Biolog GEN III plates. A principal component analysis (PCA) performed with the data obtained after 72 h of incubation revealed distinct metabolic profiles that were not associated with the substrate type used for the enrichment (bagasse or straw) (Fig. 1). Most of the variation is explained by the first axis (35.66%); however, variation is moderately explained by the second axis (17.36%). We clustered the PCA results according to the four resulting sections in the plot (I, II, III, and IV denoted by different colors in Fig. 1), thus determining four distinct FMG. We noticed that according to the nature of the Biolog substrates (Table S2 supplementary information), functional metabolic groups III and IV have a preference for carbohydrate metabolism. Groups I and II, on the other hand, have a preference for amino acids and carboxylic acids.

A total of 18 representative strains from each of the FMG (see Table 2) were then used to construct five-species MAMC, thus creating a total of 65 synthetic communities by combining members of two, three, or all four functional metabolic groups (Table 1).

**Table 2:** Selected strains from each of the four functional metabolic groups

Functional metabolic group	ID strain no.	Identification
I	76	*Cupriavidus pauculus* partial 16S rRNA gene strain KPS201
	10	*Pseudomonas plecoglossicida* strain ICB-TAB78 16S ribosomal RNA gene
	26	*Alcaligenes* sp. DA5 16S ribosomal RNA gene
	62	*Paracoccus* sp. B160 16S ribosomal RNA gene
II	49	*Achromobacter* sp. HBCD-1 16S ribosomal RNA gene
	90	*Devosia riboflavina* strain HPG62 16S ribosomal RNA gene
	57	*Ochrobactrum* sp. 71B2 16S ribosomal RNA gene
	85	*Sphingobacterium* sp. Bt-34 16S ribosomal RNA gene
III	68	*Brevundimonas* sp. R3 16S ribosomal RNA gene
	11	*Cellulosimicrobium* sp. BAB-2381 16S ribosomal RNA gene
	29	*Chryseobacterium taiwanense* strain DUCC3723 16S ribosomal RNA gene
	52	*Flavobacterium* sp. WG1 partial 16S rRNA gene strain WG1
	18	*Paenibacillus* sp. PALXIL05 16S ribosomal RNA gene
IV	41	*Enterobacter aerogenes* strain K_G_AN-5 16S ribosomal RNA gene
	44	*Pseudomonas* sp. GT 2-02 16S ribosomal RNA gene
	25	*Microbacterium* sp. UYFA68 16S ribosomal RNA gene
	15	*Bacillus nealsonii* strain RTA5b2 16S ribosomal RNA gene
	13	*Stenotrophomonas maltophilia* strain JN40 16S ribosomal RNA gene

### Degradation of Lignocellulose Fractions and Correlation with Metabolic Potentials

**Fig. 2:**
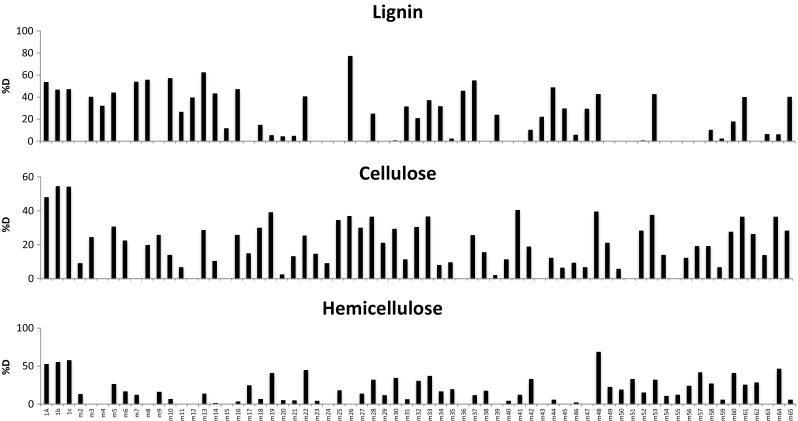
Percentage of degradation (%D) of the three lignocellulosic fractions obtained with FTIR for each of the MAMC—synthetic communities of five species (m2-m65), taken from a pool of 18 selected lignocellulose degrading strains. The first three bars (1a–1c) represent the degradation potential of all 18 strains combined

FTIR analysis performed on dried substrate obtained after consortia’s growth depicts degradation of lignocellulose per lignocellulose’s fraction (lignin, cellulose, and hemicellulose). Figure 2 shows the percentage of degradation of all 65 synthetic communities including three replicates containing all 18 strains per lignocellulosic fraction. From the results, we could distinguish that the synthetic communities type A (MAMC with four functional groups, m2-m17) degraded lignin effectively when compared to the degradation of hemicellulose and cellulose. On the other hand, type C synthetic communities (MAMC with three functional groups, m54-m65) have a better degradation of hemicellulose. A MDS (Fig. S3 Supplementary information) plotting mixes type A and type C shows a fair separation between these two types. Synthetic communities type B (MAMC with two functional groups, m18-m53), however, do not have a clear clustering since the degradation levels of all three lignocellulosic fractions were rather heterogeneous among communities (data not plotted). The synthetic community containing all 18 strains shows the highest degradation profile of all lignocellulosic fractions (52% on average).

**Fig. 3:**
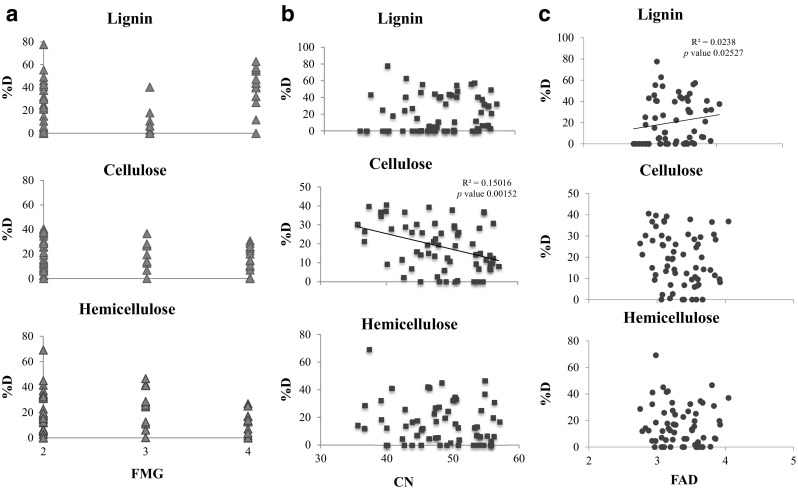
Relationship between functional diversity measurements and the degradation potential of the microbial consortia in each of the lignocellulose fraction: lignin, cellulose, and hemicellulose. **a** Degradation (%D) as a function of the number of functional metabolic groups (FMG), defined according to the metabolic potential of the strains used in the used in the synthetic communities (see Fig. 1). **b** Degradation (%D) as a function of the community niche (CN), which represents the maximum metabolic potential of the mixture [9, 10]. **c** Degradation (%D) as a function of the functional attribute diversity (FAD), calculated using the Biolog metabolic data from Biolog GEN III plates. Functional diversity measurements were calculated using the data generated by the metabolic potential of the individual strains. Only significant relationships are indicated

In order to measure the degree of functionality across the synthetic communities, and their potential effect on lignocellulose degradation, we used three FD measures: (i) FMG, which represents the number of functional groups in the synthetic community; (ii) CN; and (iii) FAD, both of which are associated with the metabolic potential of the community. Our results revealed that both the FD and the lignocellulose component influenced the relationship (Fig. 3). FMG did not generate any significant pattern, although a positive relationship between lignin degradation and FMG was close to significant (*p* value = 0.061), a positive tendency was also observed in CN results. A positive relationship was equally observed between the degradation of lignin (*p* value = 0.025) and FAD, although the explanatory power was very low (*R*^2^ = 0.0238). Conversely, higher metabolic diversity (CN) had a negative and significant effect on the degradation of cellulose and negative tendency on the degradation of hemicellulose, showing here a similar pattern than those obtained with FMG and FAD.

### Identification of Potential Strains and the Best MAMC

**Fig. 4:**
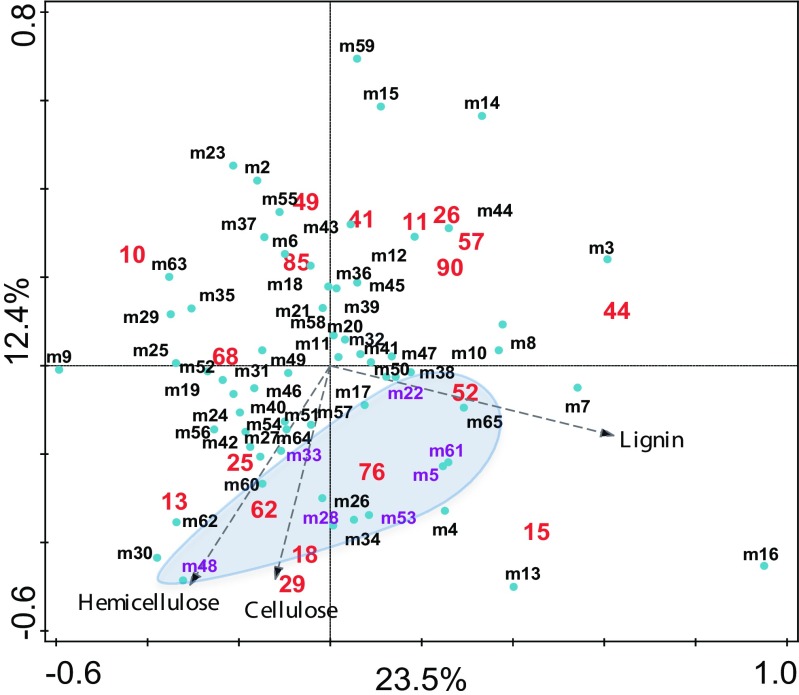
Multivariate analyses depicting the degradation potential of all MAMC. Redundancy analyses (RDA) were performed using the degradation results on all three lignocellulose components of all synthetic communities (blue circles) as well as the individual strains (as environmental data, in red). Degradation activity of MAMC is based on the FITR method whereas that from individual strains is based on MUF analyzes. Arrows indicate the activity of each lignocellulose component. An ellipse highlights the synthetic communities with > 30% degradation potential, when compared to the total amount of lignocellulose available in the sugarcane bagasse or straw

We identified a total of seven synthetic communities with an averaged degradation > 30% (maximum value 50.6%) in all three lignocellulosic fractions: m5, m22, m28, m33, m48, m53, and m61. A RDA triplot using the degradation results data vs the combination of strains (as environmental data) revealed that the seven synthetic communities with relatively high degradation clustered together and shared specific strains (Fig. 4). Table 3 summarizes the degradation results of the seven synthetic communities.

**Table 3:** The seven synthetic communities with degradation results %D > 30 and their specific degradation for each one of the 3 lignicellulose components, lignin, cellulose and hemicellulose

	Degradation (%D)		
Synthetic community	Lignin	Cellulose	Hemicellulose
m5	44.21	30.74	26.93
m22	40.79	25.51	45.09
m28	25.08	36.73	32.28
m33	37.56	36.84	37.05
m48	43.01	39.65	69.18
m53	42.76	37.83	32.38
m61	40.32	36.67	25.81

The most effective lignocellulose-degrading strains common among the seven selected synthetic communities were *Stenotrophomonas maltophilia* strain *JN40* (ID Number. 13)*, Paenibacillus* sp. *PALXIL05* (ID Number 18)*, Microbacterium* sp. *UYFA68* (ID Number 25)*, Chryseobacterium taiwanense* strain *DUCC3723* (ID Number 29*), Paracoccus* sp. *B160* (ID Number 62)*, Brevundimonas* sp. R3 (ID Number. 68), *Burkholderia* sp. *YX02* 16S ribosomal RNA gene (ID Number 76*).* According to the degradation results. the highest degradation was observed in the synthetic community number 48 (degradation averaged of all lignocellulose fractions 50.6%) containing strains with ID numbers 13, 18 25, 29. and 68. All the strains in this synthetic community belonged to the functional metabolic groups with preference for carbohydrate metabolism (groups III and IV).

## Discussion

In this study, we used a reductive-screening approach coupled with ecological strategies to obtain minimal active microbial consortia (MAMC) capable of effectively degrading lignocellulose, using bacterial strains obtained from environmental (soil) samples. Our reductive method included an enrichment experiment and the isolation of final community members. Furthermore, we used enzymatic and metabolic-profile assessments to determine the degradation potential and metabolic property of the isolated members of the community. Enzymatic assessments are rather simple, powerful, and quantitative approaches for screening purposes and have been effectively used in analyzing lignocellulose degradation capacities [26, 29, 40]. Here, we used this method for screening purposes, focusing only on enzymes from the GH family, which are related to hemicellulose and cellulose degradation. Furthermore, the use of metabolic profiling tools like Biolog was advantageous in depicting metabolic preferences of the initially selected strains, define functional metabolic groups, identify the metabolic preferences of the effective degrading bacteria, and reveal the relationship between functional diversity and degradation.

Using the metabolic and enzymatic characterization of the strains together with 16S rRNA gene sequencing, we further designed 65 MAMC (bacterial communities containing synthetic communities of only five microbial species) that were functionally diverse, i.e., displaying various levels of complementarity in terms of both carbon source utilization and enzymatic activities associated with the different lignocellulose components. These functionally complementary communities were then assessed for their capacity to degrade all three lignocellulose fractions (see Fig. S1(b) in the supplementary information for a schematic representation of the reductive screening approach). Our aim was to determine whether high functional diversity would lead to higher degradation potential, as predicted in community ecology theory [41], given that high functionality is expected to lead to higher number of (complementary) functions or ecological roles needed for an effective degradation.

Our results showed that the number of strains included in the consortia did not determine the effectivity of degradation since the results obtained with all 18 strains were comparable to the results obtained with several five-strain constructed synthetic communities (the synthetic community with the maximum averaged degradation reached up to 96.5% of the degradation rate when compared to the synthetic community containing all 18 strains). Other studies obtained similar degradation levels (between 40 and 50% average of all three lignocellulosic fractions [28, 29] with more than 20 different identified strains in the community). Here, we could identify a total of seven synthetic communities with optimal degradation levels. Results were mostly identity dependent, but there were positive and negative connections with functional diversity. We can then conclude that reducing the number of community members provides a better overview of functional diversity and ecological roles without affecting the degradation levels. In another notice, we found that higher functional diversity increased the degradation of the most complex substrate (lignin). Lignin’s breakdown involves the release of a great variety of compounds including complex aromatic carboxylic acids, which might eventually converge into the citric acid cycle [42]. Our results suggest that the strains with the enzymatic machinery able to metabolize lignin metabolic products (aromatic carboxylic acids) might also have the enzymatic means to metabolize complex substrates like lignin.

Thus, the so-called division of labor together with diversity in our built consortia might be acting beneficially upon the potential of the community during lignin degradation. Our results showed that a higher functional diversity could expand and add complementarity for resource use among taxa in the context of bacterial community functioning [38]. On the other hand, the degradation levels of cellulose negatively correlated with the diversity measures based on overall carbon metabolism (FAD and CN) whereas non-significant but negative trends were observed for hemicellulose. The enzymatic degradation of these substrates is accomplished via the collective action of multiple carbohydrate-active enzymes, typically acting together as a cocktail with complementary, synergistic activities and modes of action [6]. This negative relationship could suggest the presence of interspecific competition, which can be a stress factor for the community.

Interestingly, we found commonality in the species among the seven selected synthetic communities with the highest degradation levels, which are frequently reported as effective lignocellulose degraders. For example, *Paracoccus* sp. was found to be dominant after selection in lignin-amended lignocellulosic cultures; this suggested that *Paracoccus* sp. has an active role in lignin modification and depolymerization [2, 43]. On the other hand, *Burkholderia* sp. has been found to secrete specific enzymes capable of degrading plant cell wall components like cellulose, hemicellulose, lignin, and xylose [44, 45]. Another study found specific genes coding for catalases and peroxidases, which might contribute to the lignin degrading ability of *Burkholderia* sp. [45]. Our RDA analysis showed a positive correlation of *Burkholderia* sp. with the degradation of all three lignocellulosic fractions.

Other genera included in the most effective synthetic communities were *Stenotrophomonas maltophilia*, *Microbacterium* sp., *Paenibacillus* sp., *Chryseobacterium taiwanense*, and *Brevundimonas* sp*.* The genus *Stenotrophomonas* occurs ubiquitously in nature and species like *S. maltophilia* are often found in the rhizosphere and inside many different plant species. Interestingly, Galai et al. [46] demonstrated laccase activity in *Stenotrophomonas maltophilia*, which was able to deconstruct lignin. Hence, *Stenotrophomonas* sp. has been found as excellent candidate for biotechnological applications. On the other hand, although there are very few reports of *Microbacterium* sp. strains with lignin degradation ability, *Microbacterium* species were found to be present in the gut of the wood-infesting termites [47]. There are also reports of strains of *Microbacterium* able to degrade polycyclic aromatic hydrocarbons [48–50].

Several strains of *Paenibacillus* sp. have been found to efficiently degrade lignocellulosic fractions [51], having effective lignolytic and cellulosytic enzymes [50, 52, 53]. On the other hand, the role of *Chryseobacterium* sp. in lignocellulose degradation is still unclear [30]; however, several species of *Chryseobacterium* have been isolated from lignocellulosic substrates and as part of the degradation processes of cellulose [54]. We found here (Fig. 3) a strong correlation of cellulose degradation with the presence of *Paenibacillus* sp. and *Chryseobacterium* sp. in the consortia*.* Moreover, the presence of *Brevundimonas sp.* (catalase producers) during lignocellulose degradation has been reported in lignin-amended soils [43] and in enrichment cultures of switchgrass and corn stover [26] and also has been found to be related to cellulose degradation [55].

Here, we were able to construct minimal effective consortia from environmental samples with optimal degradation levels as the ones found in consortia built with a higher number of community members. We found that a consortium containing *Stenotrophomonas maltophilia, Paenibacillus* sp., *Microbacterium* sp., *Chryseobacterium taiwanense*, and *Brevundimonas* sp. is an effective degrading synthetic community. Further work would be needed to understand the interactive relationship and metabolic pathways of these consortium partners. However, the screening method described here demonstrated to be a useful way to obtain minimal lignocellulose-degrading consortia from environmental samples that can be further developed to efficiently promote a sustainable way of lignocellulose breakdown for environmental or commercial purposes.

## Electronic supplementary material

ESM 1 (PPTX 7.40 mb)
