# Supplementary material for: Construction of Effective Minimal Active Microbial Consortia for Lignocellulose Degradation
Source: Microb Ecol. 2018 Feb 1;76(2):419–29. doi: 10.1007/s00248-017-1141-5 (PMC6061470; doi:10.1007/s00248-017-1141-5)
Supplement: Supplementary file 1 — (PPTX 7.40 mb) [file 248_2017_1141_MOESM1_ESM.pptx]

## Slide 1
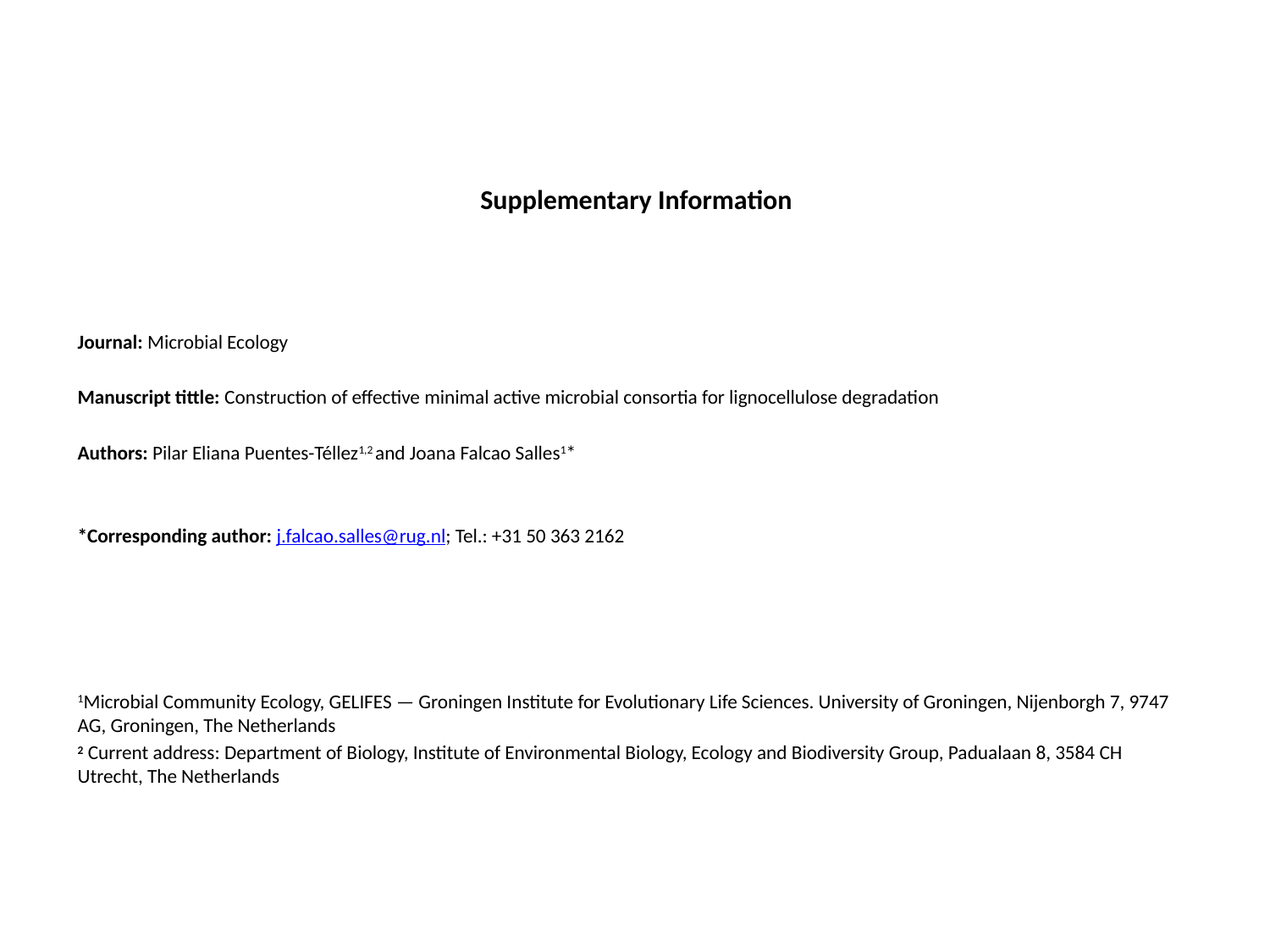

Supplementary Information
Journal: Microbial Ecology
Manuscript tittle: Construction of effective minimal active microbial consortia for lignocellulose degradation
Authors: Pilar Eliana Puentes-Téllez1,2 and Joana Falcao Salles1*
*Corresponding author: j.falcao.salles@rug.nl; Tel.: +31 50 363 2162
1Microbial Community Ecology, GELIFES — Groningen Institute for Evolutionary Life Sciences. University of Groningen, Nijenborgh 7, 9747 AG, Groningen, The Netherlands
2 Current address: Department of Biology, Institute of Environmental Biology, Ecology and Biodiversity Group, Padualaan 8, 3584 CH Utrecht, The Netherlands

## Slide 2
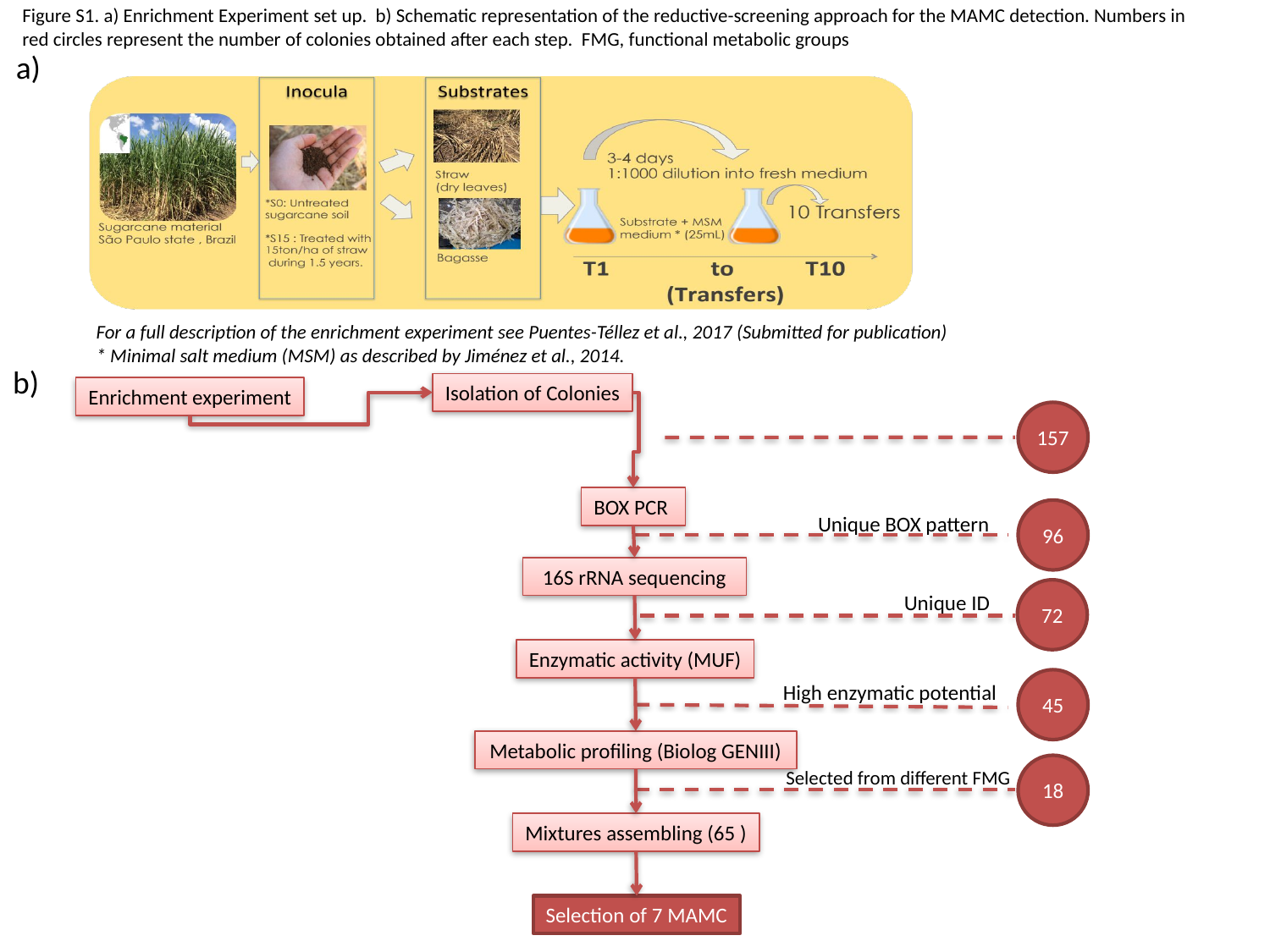

Figure S1. a) Enrichment Experiment set up. b) Schematic representation of the reductive-screening approach for the MAMC detection. Numbers in red circles represent the number of colonies obtained after each step. FMG, functional metabolic groups
a)
For a full description of the enrichment experiment see Puentes-Téllez et al., 2017 (Submitted for publication)
* Minimal salt medium (MSM) as described by Jiménez et al., 2014.
b)
Isolation of Colonies
Enrichment experiment
157
BOX PCR
96
Unique BOX pattern
16S rRNA sequencing
72
Unique ID
Enzymatic activity (MUF)
45
High enzymatic potential
Metabolic profiling (Biolog GENIII)
18
Selected from different FMG
Mixtures assembling (65 )
Selection of 7 MAMC
Enrichment Experiment /Obtaining the consortia
Obtaining the consortia
Obtaining the consortia
Obtaining the consortia
Obtaining the consortia
3-4 days
3-4 days
3-4 days
1:1000
3-4 days
Straw
(dry leaves)
1:1000
Straw
(dry leaves)
10 Transfers
1:1000
Straw
(dry leaves)
10 Transfers
1:1000
Straw
(dry leaves)
10 Transfers
10 Transfers
Sugarcane related soils
S1: No treated
S2 : (Treated with 15ton/ha of straw)
Sugarcane related soils
S1: No treated
S2 : (Treated with 15ton/ha of straw)
Sugarcane related soils
S1: No treated
S2 : (Treated with 15ton/ha of straw)
Sugarcane related soils
S1: No treated
S2 : (Treated with 15ton/ha of straw)
Sugarcane material
São Paulo state , Brazil
Sugarcane material
São Paulo state , Brazil
T1 to T10
 (Transfers)
Sugarcane material
São Paulo state , Brazil
T1 to T10
 (Transfers)
Sugarcane material
São Paulo state , Brazil
T1 to T10
 (Transfers)
T1 to T10
 (Transfers)
Bagasse
Bagasse
Bagasse
Bagasse

## Slide 3
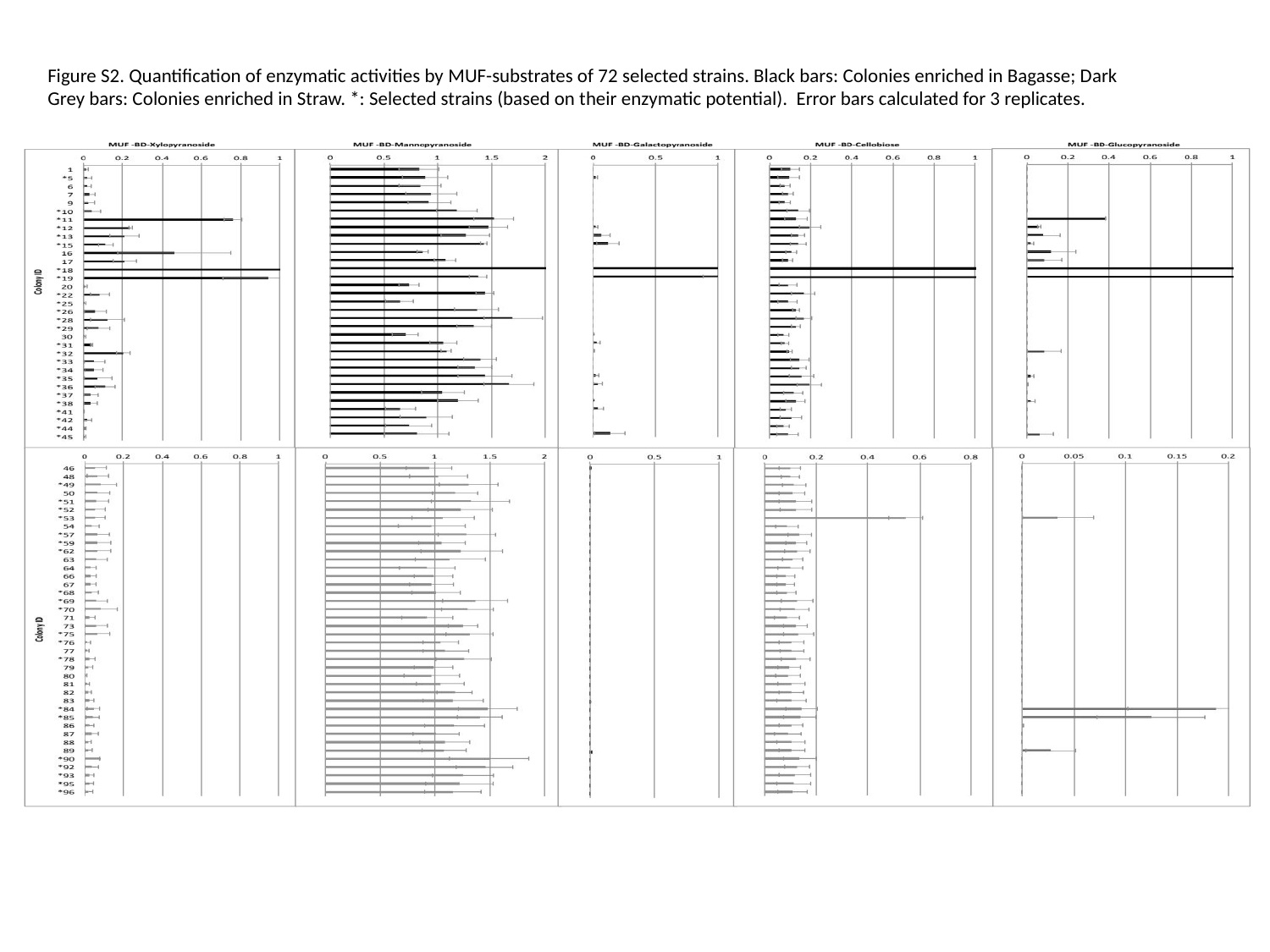

# Figure S2. Quantification of enzymatic activities by MUF-substrates of 72 selected strains. Black bars: Colonies enriched in Bagasse; Dark Grey bars: Colonies enriched in Straw. *: Selected strains (based on their enzymatic potential). Error bars calculated for 3 replicates.

## Slide 4
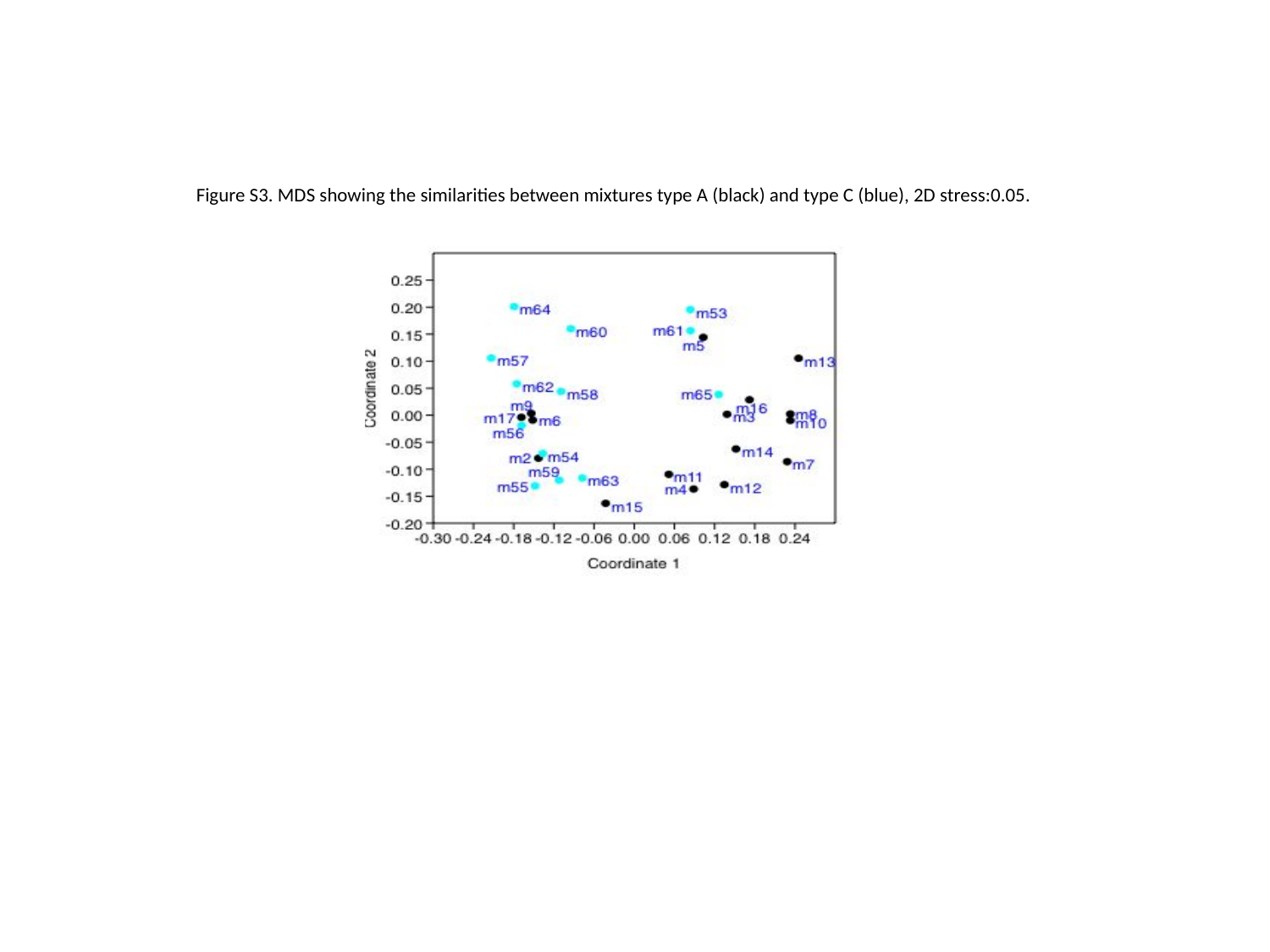

# Figure S3. MDS showing the similarities between mixtures type A (black) and type C (blue), 2D stress:0.05.

## Slide 5
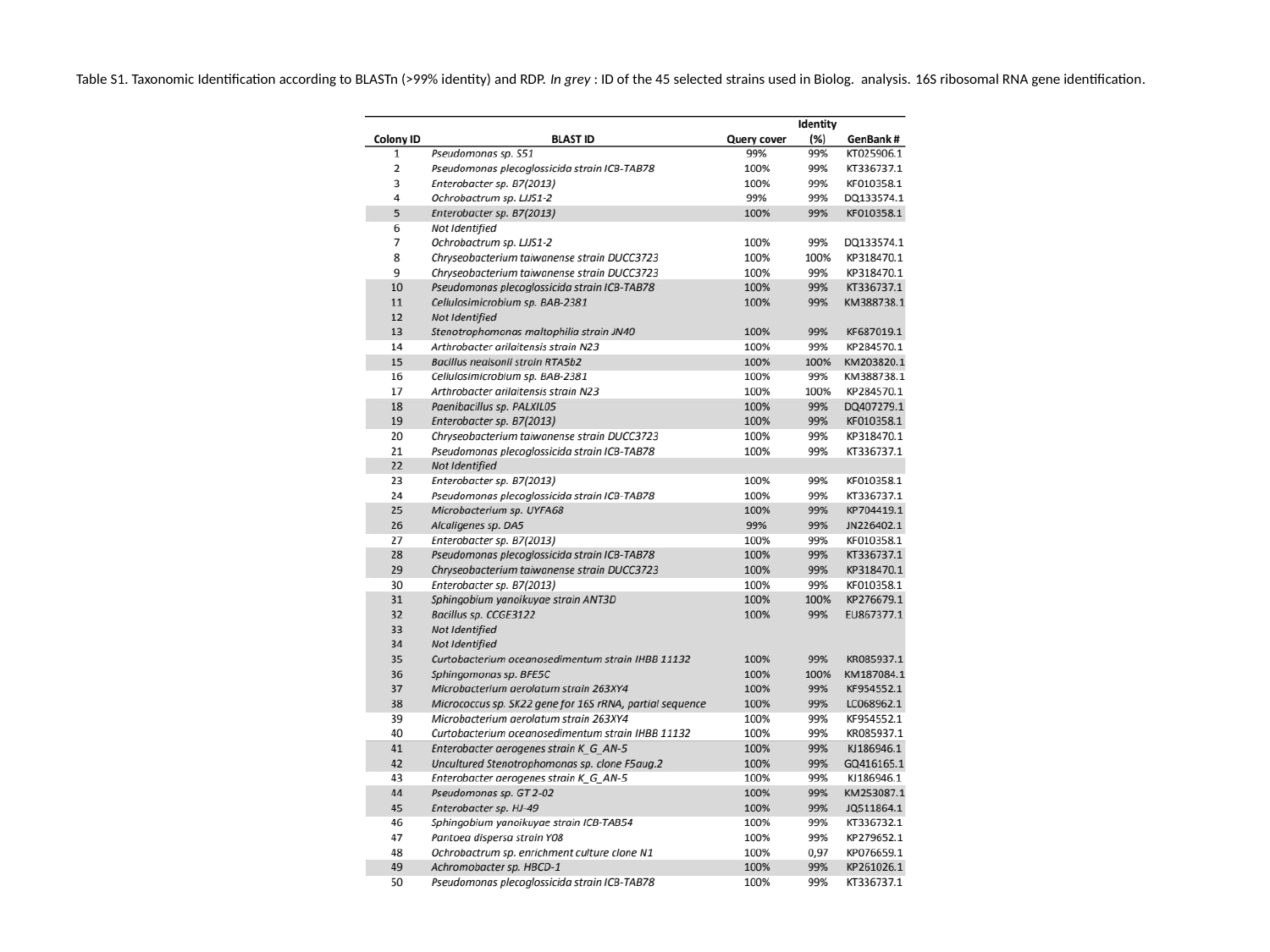

# Table S1. Taxonomic Identification according to BLASTn (>99% identity) and RDP. In grey : ID of the 45 selected strains used in Biolog. analysis. 16S ribosomal RNA gene identification.

## Slide 6
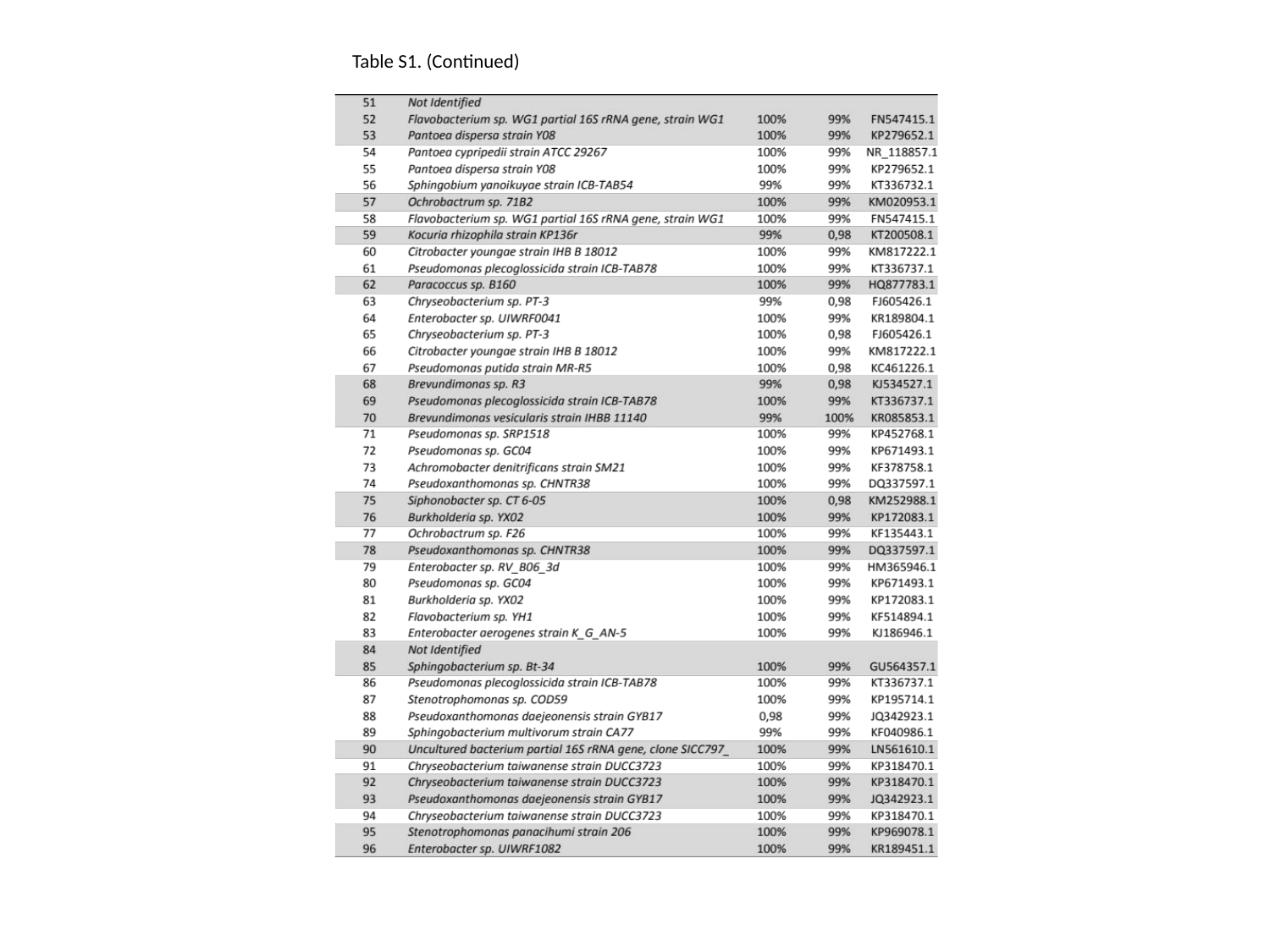

Table S1. (Continued)

## Slide 7
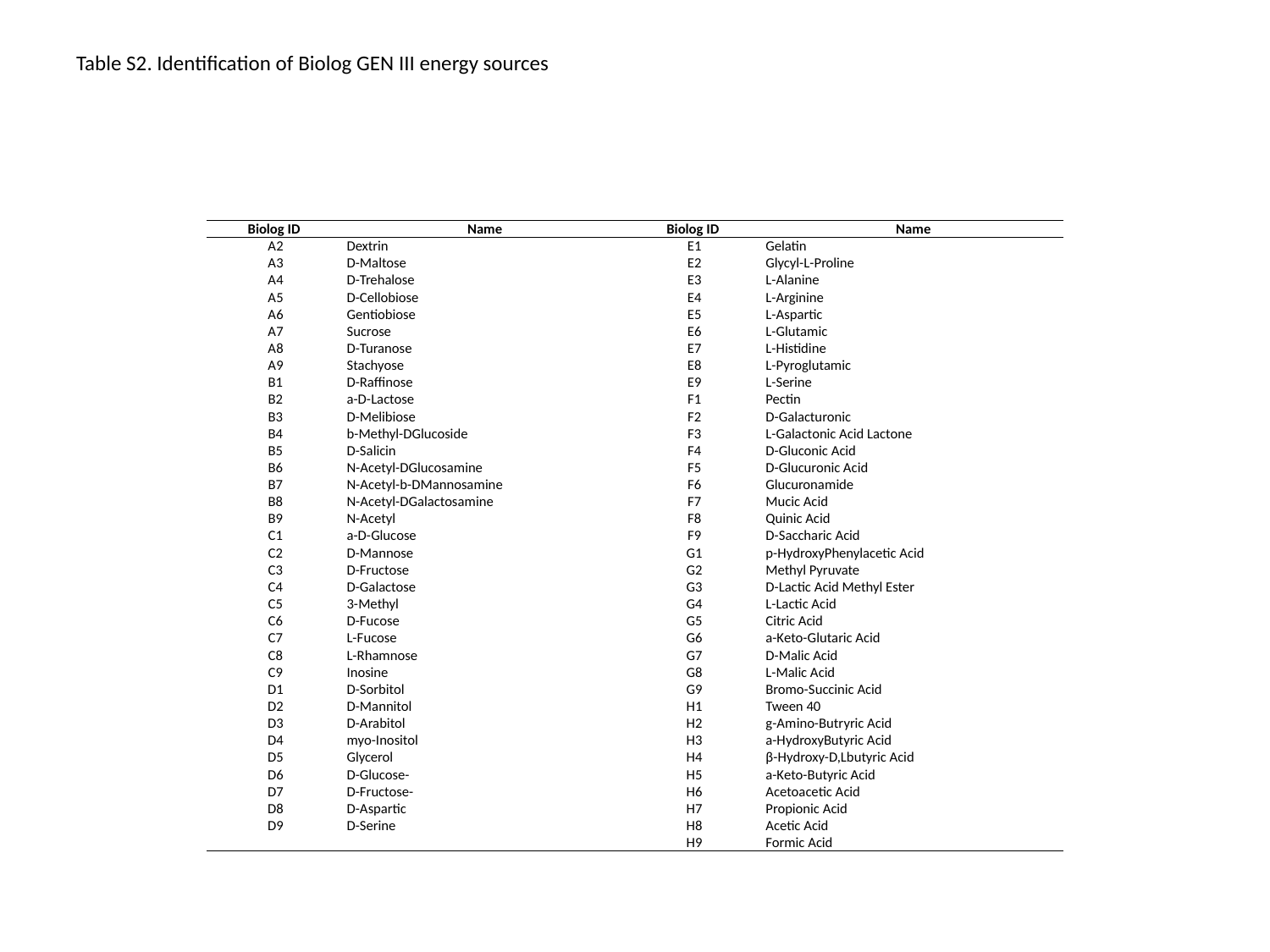

# Table S2. Identification of Biolog GEN III energy sources
| Biolog ID | Name | Biolog ID | Name |
| --- | --- | --- | --- |
| A2 | Dextrin | E1 | Gelatin |
| A3 | D-Maltose | E2 | Glycyl-L-Proline |
| A4 | D-Trehalose | E3 | L-Alanine |
| A5 | D-Cellobiose | E4 | L-Arginine |
| A6 | Gentiobiose | E5 | L-Aspartic |
| A7 | Sucrose | E6 | L-Glutamic |
| A8 | D-Turanose | E7 | L-Histidine |
| A9 | Stachyose | E8 | L-Pyroglutamic |
| B1 | D-Raffinose | E9 | L-Serine |
| B2 | a-D-Lactose | F1 | Pectin |
| B3 | D-Melibiose | F2 | D-Galacturonic |
| B4 | b-Methyl-DGlucoside | F3 | L-Galactonic Acid Lactone |
| B5 | D-Salicin | F4 | D-Gluconic Acid |
| B6 | N-Acetyl-DGlucosamine | F5 | D-Glucuronic Acid |
| B7 | N-Acetyl-b-DMannosamine | F6 | Glucuronamide |
| B8 | N-Acetyl-DGalactosamine | F7 | Mucic Acid |
| B9 | N-Acetyl | F8 | Quinic Acid |
| C1 | a-D-Glucose | F9 | D-Saccharic Acid |
| C2 | D-Mannose | G1 | p-HydroxyPhenylacetic Acid |
| C3 | D-Fructose | G2 | Methyl Pyruvate |
| C4 | D-Galactose | G3 | D-Lactic Acid Methyl Ester |
| C5 | 3-Methyl | G4 | L-Lactic Acid |
| C6 | D-Fucose | G5 | Citric Acid |
| C7 | L-Fucose | G6 | a-Keto-Glutaric Acid |
| C8 | L-Rhamnose | G7 | D-Malic Acid |
| C9 | Inosine | G8 | L-Malic Acid |
| D1 | D-Sorbitol | G9 | Bromo-Succinic Acid |
| D2 | D-Mannitol | H1 | Tween 40 |
| D3 | D-Arabitol | H2 | g-Amino-Butryric Acid |
| D4 | myo-Inositol | H3 | a-HydroxyButyric Acid |
| D5 | Glycerol | H4 | β-Hydroxy-D,Lbutyric Acid |
| D6 | D-Glucose- | H5 | a-Keto-Butyric Acid |
| D7 | D-Fructose- | H6 | Acetoacetic Acid |
| D8 | D-Aspartic | H7 | Propionic Acid |
| D9 | D-Serine | H8 | Acetic Acid |
| | | H9 | Formic Acid |
